# Overcoming burdens in the regulation of clinical research in children. Proceedings of a consensus conference, in historical context

**DOI:** 10.1186/1687-9856-2011-19

**Published:** 2011-12-30

**Authors:** Robert J Levine, Myron Genel, Leona Cuttler, Dorothy J Becker, Lynnette Nieman, Robert L Rosenfield

**Affiliations:** 1 Interdisciplinary Center for Bioethics, Yale University, New Haven, CT 06520-8293, USA; 2 Yale Child Health Research Center, Department of Pediatrics, Yale University School of Medicine, New Haven, CT 06520-8081, USA; 3 The Center for Child Health and Policy, Case-Western Reserve University, Cleveland OH 44106, USA; 4 University of Pittsburgh School of Medicine, Pittsburgh, PA 15224, USA; 5 The Eunice Kennedy Shriver National Institute of Child Health and Human Development (NICHD), National Institutes of Health, Bethesda, MD 20892, USA; 6 The University of Chicago Pritzker School of Medicine, Chicago, IL 60637, USA

**Keywords:** Institutional Review Board, ethics, pediatrics, endocrinology, federal regulations, clinical research

## Abstract

**Background:**

Many investigators are concerned that the modes of implementation and enforcement of the federal regulations designed to protect children are unduly impeding pediatric clinical research.

**Objective:**

To assess regulatory impediments to clinical research involving children and to develop recommendations to ameliorate them.

**Participants:**

The Pediatric Endocrine Society and The Endocrine Society convened a consensus conference involving experts and stakeholders in patient-oriented research involving children and adolescents in 2008.

**Consensus process:**

Following presentations that reviewed problematic issues around key regulations, participants divided into working groups to develop potential solutions that could be adopted at local and federal levels. Presentations to the full assembly were then debated. A writing committee then drafted a summary of the discussions and main conclusions, placing them in historical context, and submitted it to all participants for comment with the aim of developing consensus.

**Conclusions:**

Recommendations designed to facilitate the ethical conduct of research involving children addressed the interpretation of ambiguous regulatory terms such as "minimal risk" and "condition" and called for the development by professional societies of best practice primers for common research procedures that would be informative to both investigators and institutional review boards. A call was issued for improved guidance from the Office for Human Research Protections and Food and Drug Administration as well as for the development by professional societies of a process to monitor progress in improving human subject research regulation. Finally, a need for systematic research to define the nature and extent of institutional obstacles to pediatric research was recognized.

## Introduction: Pediatric-Specific Issues in Clinical Research

Compliance with federal regulations often impedes the transfer of basic scientific advances to the bedside [1]. Varying interpretation of these regulations by regulators, institutional administrators, institutional review boards (IRBs), accreditation agencies and investigators has exacerbated the burdens.

In conducting research to understand childhood diseases and to develop optimal treatments, protection of children is essential. However, to conduct research in children, it is often necessary to enroll both unhealthy and healthy children in clinical research that involves some risk. Pediatric investigators share with Office of Human Research Protections (OHRP), the Food and Drug Administration (FDA), and IRBs the responsibility to make research in children as safe and free from risk as it can be without forfeiting the possibility of realizing the scientific aims of the study. Regulatory burdens arise from the inherent tension between the importance of research and the need to protect children.

The Code of Federal Regulations (CFR) includes both general provisions for the protection of human research subjects (the "Common Rule", 45CFR46 Subpart A) and special provisions designed to protect children (45CFR46, Subpart D: Additional Protections for Children Involved as Subjects in Research). In the last few decades enforcement of these regulations increased appreciably and critical reports from the U. S. General Accounting office and other federal agencies were accompanied by a series of highly publicized federal shutdowns of prominent academic centers by the National Institutes of Health (NIH) Office for Protection from Research Risks (OPRR) [2,3]. In 2000, OPRR functions were strengthened and transferred to the Office of the Assistant Secretary for Health as OHRP [4]. Specifically regarding children, the *Children's Health Act of 2000 *[5-7] required all research "conducted, supported or regulated" by the Department of Health & Human Services (HHS) to comply with 45CFR46, Subpart D, and the Best Pharmaceuticals for Children Act of 2002 [8] engendered an Institute of Medicine report on oversight of clinical research involving children, including "the roles and responsibilities" of IRBs [9]. One consequence of these events is that oversight by IRBs has been heightened, though not always consistently applied, particularly where federal regulations are ambiguous [3].

Parallel regulations from HHS and the FDA categorize research that is permissible in children as follows: 1) research that does not involve greater than minimal risk (45CFR46.404/21CFR50.51) (Table 1), 2) research that includes interventions or procedures that present more than minimal risk which, themselves, hold out the prospect of direct benefit for the individual child-subject (45CFR46.405/21CFR50.52), 3) research that includes interventions or procedures that present more than minimal risk which, themselves, do not hold out the prospect of direct benefit for the individual child-subject and are likely to yield generalizable knowledge about the subjects' disorder or condition (45CFR46.406/21CFR50.53) (Table 2), and 4) research that presents greater than minimal risk which is not approvable in categories 1-3, but presents a reasonable opportunity to further the understanding, prevention, or alleviation of a serious problem affecting the health or welfare of children (45CFR46.407/21CFR50.54) (Table 3).

**Table 1:** Regulatory definition of minimal risk

45 CFR 46.102i:	"The probability and magnitude of harm or discomfort anticipated in the research are not greater in and of themselves than those ordinarily encountered in daily life or during the performance of routine physical and psychological examinations or tests."
Secretary's Advisory Committee on Human Research Protections recommendation (July 28, 2005):	For children below the age of legal consent, the standard "should be interpreted as those risks encountered during daily life by normal, average, healthy children living in safe environments...(and)...should be indexed to risks... experienced by children the same age and developmental status as the subject population."

**Table 2:** Permissible research that poses more than minimal risk without direct benefit to the child-subject

45 CFR 46.406 (FDA 21 CFR 50.53 analogous). "Research in which the IRB finds that more than minimal risk is presented by an intervention or a procedure that does not hold out the prospect of direct benefit for the individual subject...(and in which):	
(a)	The risk represents a minor increase over minimal risk;
(b)	The intervention or procedure presents experiences to subjects that are reasonably commensurate with those inherent in their actual or expected medical, dental, psychological, social, or educational situations;
(c)	The intervention or procedure is likely to yield generalizable knowledge about the subjects' disorder or condition which is of vital importance for the understanding or amelioration of the subjects' disorder or condition; and
(d)	Adequate provisions are made for soliciting assent of the children and permission of their parents or guardians, as set forth in §46.408."

**Table 3:** Conditions for federal approval of research that is not IRB-approvable according to any other regulatory standards

45 CFR 46.407 (FDA 21 CFR 50.54 analogous). "Research not otherwise approvable which presents an opportunity to understand, prevent, or alleviate a serious problem affecting the health or welfare of children. HHS will conduct or fund research that the IRB does not believe meets the requirements of §46.404, §46.405, or §46.406 only if:			
(a)	the IRB finds that the research presents a reasonable opportunity to further the understanding, prevention, or alleviation of a serious problem affecting the health or welfare of children; and		
(b)	the Secretary, after consultation with a panel of experts in pertinent disciplines (for example: science, medicine, education, ethics, law) and following opportunity for public review and comment, has determined either:		
	(1)	that the research in fact satisfies the conditions of §46.404, §46.405, or §46.406, as applicable, or	
	(2)	the following:	
		(i)	the research presents a reasonable opportunity to further the understanding, prevention, or alleviation of a serious problem affecting the health or welfare of children;
		(ii)	the research will be conducted in accordance with sound ethical principles; *and*
		(iii)	adequate provisions are made for soliciting the assent of children and the permission of their parents or guardians, as set forth in §46.408."

The terms "minimal risk", "minor increase over minimal risk", and "condition" are crucial to justifying any pediatric research proposal. However, their ill-defined nature has led to considerable variability in interpretation of the regulations. They present to investigators and all participants in the review and approval process a distinct set of challenges over and above those involved in research on adult subjects. Furthermore, research that presents more than minimal risk to normal child-subjects and no prospect of direct benefit is approvable only if it is referred by an IRB to HHS for the opportunity for public comment and for review by a panel of national experts in pertinent disciplines to determine whether it is ethical and meets the regulatory criteria for approval under 45CFR46.407/21CFR50.54. This national ethical advisory panel is commonly called a "407" panel.

The Pediatric Endocrine Society and The Endocrine Society are concerned that the modes of implementation and enforcement of these regulations are unnecessarily impeding essential research involving children. In making a decision about any particular protocol, all the ambiguous terms in the preceding paragraph must be interpreted by researchers and IRB members; there is no explicit guidance for doing so from OHRP or the FDA. The Societies hosted a workshop to address these obstacles specific to pediatric research on the 2^nd ^day of a conference on Regulation of Clinical Research hosted by the Societies on November 5-6, 2008 in Bethesda, MD. The participants were experts representative of the community of stakeholders in patient-oriented research involving children as subjects and included investigators, institutional administrators, IRB members, ethicists, and representatives from professional societies, FDA, OHRP, the Clinical and Translational Science Awards (CTSA) program, patients, and patient advocates. The goal of the conference was to find ways to facilitate the ethical conduct of research involving children. The major thrust was to develop recommendations for a) overcoming the regulatory burdens imposed by variable interpretations of the minimal risk standard and the definitions of "disorder" or "condition" and b) to facilitate the process for approval of an intervention or procedure that poses a minor increment over minimal risk for normal children. Subsequently OHRP has announced proposals to improve the rules protecting human research subjects that seeks many of the same goals, namely, to enhance protection for human research subjects while reducing burden, ambiguity, and delay for investigators [10]. It should be noted that issues connected specifically to pediatric research are not addressed by these proposals, but are left for future consideration.

## Methods

After presentations in plenary sessions by experts and general discussion of the problems in regulatory terminology and in studying "healthy" children (including those selected because they are at risk of a disorder and those who serve as "normal controls"), problem-solving workshops were held. Subsequently, following presentations of conclusions from the workshops, potential solutions were debated by the general assembly. A writing committee then drafted a summary of the discussions and main recommendations, placing them in the historical context of the regulations.

Participants were given the opportunity to comment on manuscript drafts and to approve, dissent, or abstain. Fifty-five of the 69 conference participants with current addresses (80%) responded [Appendix 1]. Of these, 28 approved, 25 abstained, and 2 dissented. All clinical investigators approved (16) or abstained due to uninvolvement (3); 8 ethicists approved, 2 abstained, 1 dissented; 11 IRB members approved, 1 abstained, 1 dissented (some attendees double-counted). Seven federal agency representatives approved; the 10 OHRP and FDA representatives abstained due to conflict of interest, though they provided technical review, and the statements and conclusions in this document do not necessarily represent their views or those of HHS. Reasons for abstention not otherwise mentioned included non-participation in pediatric workshops (9); ambivalence about emphasis (2); or unstated (3). Dissents were centered around the emphasis on problems at the IRB and federal level rather than on investigator behavior, the desire for fuller discussion of disagreements about the consensus analysis of both the minimal risk standard and the definition of "condition", the proposed IRB appeal process, and consensus recommendations to improve the 407 processes.

## Case report

This case report was presented to illustrate the delays to carefully designed research that can result from variability in interpretation of the regulations. In this case the problems arose from the lack of a uniform standard for the assessment of minimal risk and of a general understanding about what constitutes a "condition".

A multicenter national trial of oral insulin prophylaxis involving children who were high-risk relatives of patients with type 1 diabetes, identified by high titers of pancreatic islet cell autoantibodies, was designed by a NIH TrialNet subcommittee. This followed promising preliminary data on a comparable subset of subjects in an antecedent study [11]. The protocol was approved by the coordinating center's university IRB and subsequently by thirteen additional American IRBs. At a fourteenth IRB, however, the process was protracted. In spite of the site investigator's responses to the IRB review in 2007, this IRB had major persistent concerns that they could not approve research that presented more than minimal risk in children who were not ill (i.e., they did not have "a condition") and, if they indeed had a condition, the screening glucose tolerance test would be dangerous because of the high glucose load. Thus, they planned to refer the protocol to OHRP for 407 review. The site investigator then submitted a written appeal to the IRB citing two lines of evidence. First, the investigator obtained a statement from their funding NIH institute stating their interpretation of "condition", which accorded with recommendations discussed below. In addition, the investigator noted that an oral glucose tolerance test in a 35 kg child involves ingesting less glucose (61 gm) than is in a medium-size McDonald's Hi-C orange drink (64 gm). The IRB ultimately withdrew its request for a 407 review and approved the study 6 months after the initial submission. After substantial disruption at the local site, participation in the national trial was eventually accomplished.

## Regulatory issues hampering translational research in children

### 1. Ambiguities in the regulatory definitions of "minimal risk" and "minor increase over minimal risk"

#### Brief historical background

The National Commission for the Protection of Human Subjects of Biomedical and Behavioral Research stated that the principle of beneficence consisted in two ethical imperatives: "do no harm" and "maximize possible benefits and minimize possible harms" [12]. They subsequently promulgated the standard for minimal risk as that "normally encountered in the daily lives, or in the routine medical or psychological examination of healthy children" [13].

The Common Rule's definition of minimal risk, (Table 1) [5,7] derived from the National Commission's recommendations, has been widely endorsed by national and international organizations, though not without controversy [14]. However, the thresholds for determining what constitutes minimal risk remain undefined in the regulations. The regulations intentionally placed the responsibility for the determination of the acceptable risk level for a research intervention or procedure on each IRB. Although true to the National Commission's commitment to local control, this approach promotes variation in application of regulations and leads to variation in the capacity of researchers to participate in well-designed and important multi-center research in children and adolescents.

Subsequently the Secretary's Advisory Committee on Human Research Protections (SACHRP) made recommendations for the interpretation of this regulatory language [15] including the adoption of a "uniform standard" for defining "minimal risk" -- that of normal, average, healthy children living in safe environments; they further recommended that evaluation of minimal risk should be indexed to risks in daily life and routine medical and psychological examinations experienced by children the same age and developmental status as the subject population (Table 1).

The National Human Research Protections Advisory Committee (NHRPC), the SACHRP predecessor, proposed examples of equivalence of risk posed by particular procedures (Table 4) [16], and SACHRP subsequently provided congruent examples of minimal risk equivalents [15]. These examples addressed the nuances of specific situations in a general manner: for example, in placement of an indwelling peripheral venous catheter, the level of risk was considered dependent on other factors, such as age of the child, the duration of catheter placement, number and volume of samples, the setting in which the procedure is performed and the experience of the operator.

**Table 4:** Examples of estimates of procedural risks

Procedure	Minimal risk	Minor increase over minimal	More than minor increase over minimal	Comment
Venipuncture	X			

Indwelling peripheral venous catheter	X			Risk level may be raised by other factors

Wrist-hand x-ray	X			

Bone density test	X			

Oral glucose tolerance test	X			

Skin punch biopsy with topical pain relief		X		

Organ biopsy			X	

MRI, no sedation	X			Risk level may be raised by other factors

MRI, with sedation		X		Intubation may decrease risk for certain children

Abridged from NHRPAC (2002).

Nevertheless, interpretation by IRBs of what tests constitute a minor increase over minimal risk is notoriously variable [17]. A survey of IRB chairs provided evidence for both over- and under-protection. For example, a single venous blood draw was categorized as minimal risk by 81% of IRB chairs, greater than minimal risk by 19%. A magnetic resonance scan without sedation was considered to constitute minimal risk by 48%, a minor increase over minimal by 35%. A weekly blood draw of 10 ml for 6 months was considered to constitute minimal risk by 15%, a minor increase over minimal by 51%. A pharmacokinetic study (risk of death: 1/100,000) was considered as minimal risk by 7%, a minor increase over minimal by 30%. Initial testing of a drug in children found safe in 500 adults was considered minimal risk by 5%, a minor increase over minimal by 23%. In each of the above cases, the remainder considered the risks to be more than a minor increase over minimal. Participant payment was incorrectly considered a direct benefit by 10%.

If an IRB finds that elements of the research pose more than minimal risk, it must decide if they are approvable because they have the potential to directly benefit the child (45CFR46.405/21CFR50.52) or meet the criteria of the other CFR sections discussed below.

#### Discussion of the regulatory minimal risk standard

Ethical considerations seldom (if ever) permit research in which one deliberately inflicts harm on a particular subject no matter how great the intended benefits. Nevertheless, there are well-established criteria for ethical justification of a statistical probability of harm. Standards set forth in 45CFR46.405/21CFR50.52 specify, for example, that the hazards associated with the development of cancer chemotherapies are justifiable by anticipated direct benefit to the subject [18]; however, such justification may be difficult to support in phase I oncology studies. 45CFR46.406/21CFR50.53 permits and specifies the conditions for justification of the imposition of a statistical possibility of harm to child-subjects for the benefit of their class of children. In the interpretation of these criteria, IRBs generally require that either the probability or magnitude of harm (preferably both) be small.

It is generally agreed that the Common Rule definition of minimal risk is ambiguous and that there is evidence for a substantial degree of inconsistency across IRBs in its interpretation [14]. The conferees concluded that revising the definition in federal regulations would be not only difficult, but potentially counterproductive; the ambiguity guards against the risk of inflexible interpretation and the politicization that could be engendered by an effort to amend the Common Rule by legislation with more precise but constrained definitions.

Therefore, the challenge is to find ways to improve implementation of the existing regulations. The experience in this era of multicenter trials, exemplified by the case report, suggests that more uniformity in IRB implementation of the regulations would be desirable. As we strive to develop the desired uniformity, we must take steps to ensure that the uniform interpretations and their implementations are transparent and reflect the views of the patient community as well as the carefully considered judgments of appropriate experts who are fully familiar with the field and the problems it faces. We must also be on guard to prevent excessive rigidity which might tend to stifle investigators' and IRBs' efforts to develop creative solutions to difficult problems.

NHRPAC and SACHRP recommendations for the interpretation of the minimal risk standard include recognition that the local context in which research is conducted--the expertise and experience of the investigator, as well as the depth and track record of expertise at the site--are important determinants of risk [19,20].

While the standard (Table 1) directs IRBs to compare the "probability and magnitude of harm or discomfort anticipated in the research...to those ordinarily encountered in daily life", a data-driven, evidence-based assessment of relative risk, as advised by SACHRP [15], does not often seem to have been adopted as the basis of decision-making, as exemplified by the case report and evidence from analysis of national advisory panel reviews [21]. Furthermore, it seemed to many participating investigators as if 'the discomforts of daily life' are underestimated by IRBs, which leads to overestimates of risk. "Discomfort" generally differs from "harm" with respect to duration, cumulative characteristics, and irreversibility [15,22]. Discomfort does not usually involve irreversible damage/injury or protracted/severe pain. Thus, discomforts seldom pose more than minimal risk of harm. The discomforts ordinarily encountered during childhood in optimal environments can be considerable (bad colds and gastroenteritis, for example).

SACHRP has also recommended that the evaluation of minimal risk should be indexed in equivalency to risks experienced by children the same age and developmental status as the subject population [15]. As the child gets older--from 8 to 12 years, for example--there are at least two relevant developments [23,24]. 1) His or her ability to perform the cognitive procedures necessary to give assent increases; there is maturing of the ability to engage in concrete and abstract reasoning and there is development of a sense of privacy that increasingly resembles that of an adult. 2) The child increasingly engages in activities that move the boundary of what counts as, or exceeds, minimal risk. An extension of this concept is that adolescents' advancing capacity for and experience with mature decision-making, for example, in health matters and understanding of altruism, gives them the potential to give the ethical equivalent of informed consent, so consideration should be made for allowing them to participate in certain types of research without permission of parents or guardians [20,25]. Thus, it seems reasonable to conclude that developmental- and age-appropriate assent parameters should allow children, particularly adolescents, to participate in individual decisions about the degree of risk that they would be prepared to tolerate in relation to the anticipated benefit they perceive in participating in approvable research. While adolescents choose more risky behaviors than adults do, which may be a reason for pause regarding adolescents, minimal risk should be evaluated in the light of risks to which the child/adolescent ordinarily is exposed and the varying autonomy that adolescents are ceded. Defenses against choices of excessively risky activities in research include the requirements for IRB review, for parental or guardian permission and, in the absence of permission by parents or guardians, the requirement by 45CFR46.408(c) of a protective mechanism comparable to that ordinarily afforded by such permission [6]. The regulations do not suggest any such protective mechanisms; however, the National Commission offered several suggestions, for example, the appointment of "a social worker, pediatric nurse or physician to act as a surrogate parent..." [13].

The provision in 45CFR46.408(c) that parental or guardian permission may be waived "if the IRB determines that a research protocol is designed for conditions or a subject population for which (such permission) is not a reasonable requirement" reflects recommendations of the National Commission [13]. The regulation names only one of the several circumstances mentioned by the National Commission for which such waivers would be appropriate--viz, "neglected or abused children". Among the other circumstances mentioned by the National Commission - one that is widely recognized by IRBs - is "[r]esearch designed to identify factors related to the incidence or treatment of certain conditions in adolescents for which, in certain jurisdictions, they may legally receive treatment without parental consent...."

In the time of the National Commission's discussions of "diseases for which adolescents may obtain treatment without parental permission," there was focus on more or less specific diseases or conditions, such as sexually transmitted diseases, drug abuse, and issues of birth control. Subsequently, many states have enacted laws permitting qualifying adolescents to receive care independent of parental permission and, in most cases, parental knowledge. This development expanded greatly the category of diseases or conditions for which adolescents may obtain treatment without parental permission. For many adolescents, this category of their activities includes most or all of their health care services [25,26]. However, federal research regulations do not preempt state consent laws, and the "mature minor" doctrine is not settled law in all states. Likewise the legal limits on a parent's ability to consent to non-beneficial, more-than-minimal risk research are not resolved in some states, such as Maryland as evidenced by the Grimes vs Kennedy Krieger Institute case [27]. As a result, each institution must determine what limits are consistent with its state law.

Investigators are concerned that, in enforcing the regulations for obtaining permission from parents and assent from children (45CFR46.408), IRBs tend to overprotect children from risk because of concern about child exploitation, by parents as well as investigators, and place insufficient emphasis on the importance of assent. This propensity of IRBs may inflate the estimation of risk and thus hinder participation in studies by children who may be mature enough to reasonably assess risk. The proper role of the IRB must be to objectively determine the upper level of risk in a protocol that will be treated as minimal for children in particular age groups and ensure that there is nonbiased representation of risk so as to avoid pressure from parents or investigators to either participate or not participate in the research. IRB approval then allows children to assent or dissent after participation in the discussion about whether the proposed risk is acceptable.

Assent is the child's affirmative agreement to participate in research. Of course, this decision is influenced by parental attitudes, and the child does not have the ultimate authority to decide whether to accept or decline. To the extent that the child has the capacity to participate, her or his opinions should be given proportional weight. While a four-year old's cognitive development does not allow many of the operations needed for assent, they are capable of choosing not to participate. Furthermore, a child's actions that rescind assent and/or otherwise constitute "dissent", i.e., deliberate objection, based on their changing evaluations of the acceptability of the risk, are clearly recognizable and are decisions that must be honored [28].

The general consensus was that HHS acceptance of NHRPAC and SACHRP recommendations for interpretation of the minimal risk standard would be an important step toward implementing a systematic method for IRB decision-making.

The conferees discussed the development by national subspecialty professional societies of "best practice primers" for common research procedures that would be informative to both investigators and IRBs to facilitate systematization of their estimates of risk. Initial steps in developing these primers should be circumscribed, however, to avoid premature expansion that would require societies to develop elaborate support infrastructure.

#### Recommendations regarding interpretation of the minimal risk standard

1. The conferees support and endorse the "daily life" and "routine medical or psychological examination or tests" Common Rule threshold as the criteria for estimating minimal risk for both harm and discomfort.

2. Investigators bear the responsibility to brief IRBs with facts and arguments to support a regulatory assessment of the level of risk and benefits in spite of the ambiguities in the regulatory definition of minimal risk.

3. To improve IRB implementation of the regulations, the conferees recommend that OHRP/FDA adopt the SACHRP recommendations as guidance for defining "minimal risk" and "minor increase over minimal risk", and recommend publishing examples of procedures or interventions that meet these criteria.

4. Professional societies should develop "best practice primers" for common research procedures suitable for use by both investigators and IRBs that address both the proper performance and degree of medical risk posed by procedures.

5. Individual children should be allowed the opportunity to participate in deciding upon involvement in a particular procedure or intervention in accordance with their developmental age, subject to parental acquiescence or refusal.

### 2. Burdens arising from ambiguities in the interpretation of what constitutes a disorder or condition (re 45CFR46.406/21CFR50.53, Table 2)

#### Brief historical background

Under the "406/53" category of the federal regulations, non-beneficial interventions or procedures involving a minor increase over minimal risk may be carried out in child subjects with a disorder or condition if they are likely to yield important generalizable knowledge about the subjects' disorder or condition, and other specifications are also satisfied (Table 2) [6]. This regulation is based on the premise that some such research can be justified ethically notwithstanding potential risks [12,18]. While it would be desirable not to expose children to any risk of research, research on childhood disorders or conditions cannot by its very nature be conducted on adults. Without such research, children would be left with an uninformed, static standard of care. The leading international documents on the ethics of medical research endorse the concept that "low risk" research is permissible in children under these circumstances, though variations in defining the permissible burden of risk reflect the underlying complex ethical issues [29-32].

The term "condition" is even less well defined than "minimal risk." The federal regulations offer no definitions of either "condition" or "disorder." The regulations on conditions stem from the 1977 National Commission report that "it is necessary to learn more about normal development as well as disease states in order to develop methods of diagnosis, treatment and prevention of conditions that jeopardize the health of children, interfere with optimal development, or adversely affect well-being in later years" [13]. Because this report did not define "condition," there is continuing controversy about whether a condition includes predisposing factors such as obesity, genetic risk, or vulnerability as part of a population at risk.

NHRPAC subsequently proposed that, "a condition relates to a specific characteristic which describes a group of children, or to a physical, social, psychological or neurodevelopmental condition affecting children, or to the risk of certain children developing a disease in the future based on diagnostic testing or physical examination" [16]. NHRPAC provided the following as examples of conditions: infancy or adolescence (i.e., normal developmental periods of a child's life); socioeconomic circumstances (e.g., poverty and institutionalization); and a specific deviant property (e.g., genetic) that would predispose the individual to the subsequent development of a disease.

However, SACHRP endorsed the Institute of Medicine 2004 advice [33] that "condition" be interpreted slightly more specifically in determining whether proposed research involving a minor increase over minimal risk and no direct benefit can be approved under 406/53. They recommended that "the term condition should be interpreted as referring to a specific (or a set of specific) physical, psychological, neurodevelopmental, or social characteristic(s) that an established body of scientific or clinical evidence has shown to negatively affect children's health and well-being or to increase their risk of developing a health problem in the future" [15]. Neither HHS nor FDA has issued guidance on this definition.

#### Discussion of defining "condition"

The definition of "condition" is variously interpreted in the absence of specific guidance, as exemplified in the case report about children at risk for type 1 diabetes. Scientific progress is identifying an increasing number of conditions as posing significant risk for the development of a health problem (e.g. genetic variants, obesity). The SACHRP definition is seen by investigators as acceptable providing that "shown" be interpreted as "indicated" so as to avoid the excessively high bar that might be imposed before risk can be conclusively proven.

While both NHRPAC and SACHRP recognized there are times when a cohort of normal healthy children may be considered as having a condition appropriate for research under 406/53 because they are part of a vulnerable population, NHRPAC's wording is somewhat broader. Infancy and adolescence are vulnerable developmental periods in many areas (e.g., neuropsychological, endocrinological). SACHRP provided the example of vaccine studies for common childhood diseases, where healthy children are at risk. Subsequent studies of human papillomavirus vaccine in healthy adolescents were thus in harmony with SACHRP recommendations. An analogous example in endocrinology would be hormonal studies that involve novel testing protocols in normal adolescent girls to understand the basis of the high prevalence of adolescent menstrual dysfunction that may have long-term health consequences [34].

#### Recommendations regarding interpretation of "condition"

1. To improve implementation of the regulations by IRBs, the conferees recommend that OHRP/FDA essentially adopt the SACHRP interpretation of "disorder or condition". This would clarify that considerations such as obesity or heritable risk factors for the disorder under study are conditions relevant to an otherwise healthy child-subject's current or future well-being and that a cohort of normal healthy children may be considered as having a condition appropriate for research because they are part of a vulnerable population.

### 3. Burdens arising in the approval process for an intervention or procedure that poses greater than minimal risk for normal children with no prospect for direct benefit (re 45CFR46.407/21CFR50.54, Table 3)

#### Brief historical background

Interventions and procedures that present more than minimal risk and no prospect of direct benefit to children who have no disorder or condition (as defined previously) are regulated according to the "407/54" category (Table 3). As noted earlier, such research was a major focus of this conference. Research in normal children is sometimes required to generate control or normative data. This regulation is based on the premise that such research can sometimes be ethically justified [12,18].

**Figure 1:**
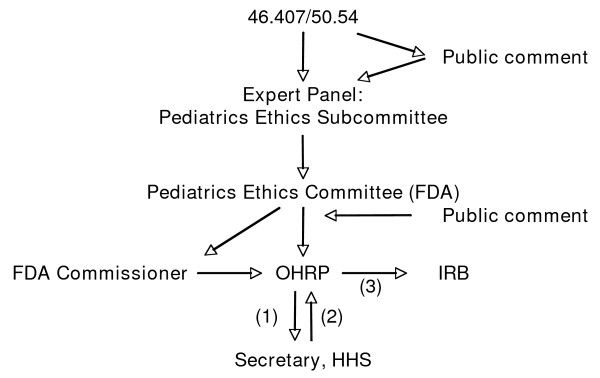
**Process for OHRP-FDA joint review of "407" referral (under 45CFR 46.407/21CFR50.54)**. A referral involving an FDA-regulated product is submitted to OHRP, which convenes a 407 panel, meanwhile posting the submission for public comment. The Pediatric Ethics Subcommittee of the FDA Pediatric Advisory Committee reviews the submission and, in a public forum, makes a recommendation to the FDA Pediatrics Advisory Committee. The recommendation of the latter is forwarded to the FDA Commissioner and OHRP, which submit their recommendation to the Secretary, HHS for approval (1), who then (2) directs OHRP to provide feedback (3) to the IRB. If OHRP feedback involves stipulations, the IRB responds directly to OHRP.

If U.S. Department of Health and Human Services funds are involved, IRBs are required to refer protocols in this category to HHS for review by a federally-convened 407 panel to ensure that any research carried out under this category is ethical. If supported by other federal agencies or a non-federal sponsor, OHRP recommends that the institution carry out a similar process for expert review that includes the opportunity for community comment. This review process was shortened to its current form in 2003, and OHRP and FDA guidance on this topic was issued in 2005-2006 (Figure 1) [5,35].

Participation in research by normal children that does not confer direct benefits may confer indirect benefits. Ackerman suggests that one indirect benefit of involvement of normal, relatively mature children in research is raising the child with social awareness -- one who would want to contribute to the well-being of the community [36]. Such research participation has been likened by McCormick to an act of charity that one should perform when one can do so without bearing too great a burden [37]. Wendler and Jenkins argue that such participation differs from usual acts of charity in that it is sacrificial, i.e., something is being done to the child, not by the child [38]; the fact remains that the child's assent is a charitable act which is "done by the child". While the child may consider money to be a benefit of participation in research, OHRP prohibits IRBs from considering payment as a direct benefit of the research when assessing the balance of risks and benefits.

When presented with a protocol in which greater than minimal risk is to be presented to an apparently normal child, an IRB must make two determinations. First, it must determine that the research is not approvable under §46.404/§50.51, 405/52, or 406/53. If an IRB considers the proposal unethical, it is expected to disapprove the protocol. If the IRB finds the minimal risk nature of the research to be unclear or determines that there is greater than minimal risk and it deems the research to be ethical and important, it must refer to an HHS 407 determination process for approval.

If an FDA-regulated product is involved, even if being used according to the label, this 407 review is conducted by the Pediatrics Ethics Subcommittee of the FDA Pediatric Advisory Committee. Because the regulation calls for the "opportunity for public review and comment", these reviews have been conducted in a public forum although this is not a requirement. This Committee then makes its recommendation to the FDA Commissioner and the Secretary of HHS for a determination.

Between 1991 and 2010 there were twenty two 407 referrals, of which 17 involved normal child subjects in varying roles in the research design, including in a control group, as healthy donors, and as healthy volunteers. Six of the 22 studies have been approved, 4 disapproved, and 12 withdrawn. Reasons for withdrawal were diverse: reconsideration by IRBs resulted in determinations that the research was approvable under 45CFR46.404, 46.405, or 46.406 (n = 4); the investigator decided not to pursue the study (n = 6), time in review playing a role in some of these decisions; or the study site was dropped from a multi-center study (n = 2). Only 7 of the 22 referrals have occurred since 2005 under the current review system: 4 of these were withdrawn, 3 approved. Median time from submission to OHRP until approval with stipulations was 10.2 months (range 8.3-10.9); median time until final approval was another 7.75 months (5.2-11.9).

#### Discussion of challenges posed by the 407 process

The challenge for institutions and IRBs is how to enable important research that will advance science and combat disease to move forward in a timely manner, while addressing the regulatory requirements. This involves keeping in mind the primacy of protecting children, while avoiding the overprotection that stymies appropriate research. Scientific advancement requires the ongoing development of control/normative data. The 407 process of approving studies in normal children can take years for a relatively straight-forward case, with about one-third of this time being spent in federal review and two-thirds in local review [39]. The conferees are concerned that investigators avoid proposing such research in children because 407 referral is such a daunting and time-consuming prospect, during which funds and resources can evaporate.

A major cause of the delay is uncertainty about what constitutes a minor increase over minimal risk on the part of both investigators and IRBs. It is incumbent upon both investigators and IRB members to become knowledgeable about the regulations. Too often, investigators fail to make a specific argument as to whether an intervention presents minimal risk or minor increase over minimal risk. Too often, IRB members are ill-prepared to render opinions based on scholarly familiarity with the regulatory issues involved [40]. Thus, it is important that investigators' protocols include IRB-targeted arguments in IRB-relevant language about risk and that IRB members be thoroughly familiar with the regulations and their implications.

The local delay stems in part from IRB tendencies toward over-protection in categorizing risk in children [21]. Differences in perception of risk cause disagreements between the IRB and investigator about the need for the 407 process, as in the case report. In part, IRB conservatism is thought by the conferees to result from overzealousness prior to facing the intense OHRP scrutiny that comes with the 407 process. IRBs and the institutions that they serve operate in defensive mode partly as a reaction to the history of punitive actions by OHRP and its predecessor, the criteria for which remain unclear. Since the 407 process itself can be streamlined very little unless redesigned, it behooves IRBs that face the prospect of 407 referral to consult with OHRP promptly to minimize the time to referral, rather than to try to first perfect their paperwork.

Part of the local delay relates to IRB members' uncertainty about risk/benefit categorization because they often lack expertise on which to make an accurate judgment of risk of harm and tend to underestimate the ordinary risks and discomforts of daily life. A single source of data about 407 outcomes in summary form to which IRBs can turn for precedent, analogous to a "case law" approach, would be helpful to IRBs and investigators. While the OHRP website provides links to the details of protocols reviewed since 2002 [5], there is no executive synopsis that abstracts the critical research procedures with a summary of the conclusions about the degree of risk they pose and the context in which they were found approvable. Such a synopsis would be an important educational tool. A model for this kind of activity was presented by the publication of case studies as a regular feature in the first several years of publication of *IRB: A Review of Human Subjects Research*.

One model to reduce such delays could be development of regional or national pediatric IRB expert consultative panels to address the challenge of assembling appropriate pediatric expertise in a single institution, especially when the only expert available is one of the investigators, whose participation in the ethical review must be limited [41]. These consultative panels would likely be "virtual", convened upon demand and constituted with sufficient pediatric and scientific expertise to render an opinion on a proposed protocol upon referral from the institutional IRB. Their authority would be limited to advisory unless the necessary paperwork were accomplished to include them in a Federal Wide Assurance as IRBs and to establish the required inter-institutional agreements. Another option could be provision by professional subspecialty societies of expert review panels and the development of the aforementioned "best practice primers" for common research procedures.

A possibility to facilitate 407 reviews for federal grant applications, which already involves a prolonged process, would be to link them to the federal scientific grant review process. While the study sections have the scientific expertise and are required to assess risk and approve the appropriateness of all human research, most of them are not broadly constituted with expertise in interpreting ethical standards or the regulations on which they are based, nor do they have the regulatory authority to serve as ethics review panels [28]. A workable system might involve sequential review, with the ad hoc addition of specific ethical and regulatory expertise on the scientific review panels, and a representative of the scientific review panel on the subsequent federal ethics panel.

Several investigators voiced concern about the adversarial attitude of IRBs and expressed their opinion that those that do not proactively communicate directly with investigators as a matter of local institutional policy are not receptive to alternative conclusions about risk. This can lead to misclassification of children as requiring 407 review, as in the case report, or it can lead to inappropriate rejection of studies of normal children and refusal to refer for 407 review. There is no widely accepted mechanism by which an investigator can challenge an IRB's disapproval. An "appellate IRB process" has been proposed to respond to the need for due process for rejected applicants [40]. Guidance from OHRP as to the desirability of direct communication with investigators and the availability of appellate mechanisms would be helpful to many investigators. Institutions are unlikely to change such policies without guidance from a regulatory agency.

The 407-approval mechanism also was noted to contain both procedural and interpretive ambiguities that raise ethical concerns, namely about the scope of the information offered to the public for comment and its potential conflicts with investigators' intellectual property or commercial interests. Also unclear is whether there is any upper limit to permissible levels of risk for interventions that do not hold out the prospect of direct benefit.

#### Recommendations regarding improving the "407" process

1. OHRP/FDA should provide executive summaries of 407/54 determinations.

2. Specialty societies should establish summary statements of procedures considered to be of minimal risk to child subjects as established by precedent and data regarding risk.

3. OHRP/FDA should provide guidance that encourages direct discourse between IRBs and investigators and that supports the availability of institutional appeal processes.^1^

4. OHRP/FDA should provide guidance for the development of a national process analogous to the federal judicial system by which investigators can appeal an IRB decision, once local mechanisms have been exhausted. This would provide recourse for investigators who believe that risk to children has been improperly categorized. Examples would include concerns about IRB inability to provide adequate scientific review, misclassification of children at risk as requiring 407 reviews, or refusal to refer for 407 reviews.

5. OHRP/FDA should encourage IRBs to actively interact with them to minimize paperwork and facilitate consideration of protocols for which 407 review is being considered.

6. Where applicable, federal granting agencies should consider means of coordinating 407 regulatory review with the federal scientific review process.

## Conclusions: Summary of recommendations for overcoming regulatory burdens in research involving children

Regulatory burdens hampering translational and other research in children arise from two major ambiguities in the terminology of the regulations. These are: 1) the ambiguities defining "minimal risk" and a "minor increase over minimal risk", and 2) ambiguities in defining a "condition" or a "disorder" and problems with interpretations of the regulations by IRBs.

These ambiguities lead to a protracted approval process for interventions or procedures judged to pose more than minimal risk for normal child research subjects. They cause different IRBs to decide similar cases differently. This is a particular problem for multicenter studies that must get approval from multiple IRBs.

These problems have been recognized for years. The Secretary's Advisory Committee on Human Research Protections (SACHRP) has built on the National Human Research Protections Advisory Committee (NHRPAC) and Institute of Medicine conclusions on this matter to issue content for recommended guidance in 2005; it transmitted its proposal to the Department of Health and Human Services (HHS) Secretary, who, in turn, recommended moving forward. Now, over 5 years later, the Office for Human Research Protections and the Food and Drug Administration (OHRP/FDA) have not issued explicit guidance that would directly implement these guidelines.

We recommend that federal agencies adopt these standards as guidance to allow more consistent interpretation of the regulations. This will not accomplish total consistency among IRBs, but would provide a common basis for decision-making. Professional societies can aid in this process by developing "best practice primers" for common research procedures suitable for use by both investigators and IRBs that address both the proper performance and degree of risk posed by commonly used procedures.

We also recommend that IRBs avoid considering children as a single group and instead recognize the increased experience, understanding and self-determination in older children and adolescents. This process, along with alleviation of other IRB procedural burdens, could be facilitated by explicit OHRP/FDA guidance.

Finally, we recognize that most of concerns about the nature and impact of regulatory burden are based on the opinions of investigators and that very limited data are available to support these opinions. Therefore, we call for research to assess the nature and extent of institutional obstacles to pediatric research and monitoring of progress in improving the balance in human subject research regulation by the stakeholder societies.

## Abbreviations

CFR: Code of Federal Regulations; FDA: Food and Drug Administration; HHS: U.S. Department of Health and Human Services; IRB: Institutional Review Board; NHRPAC: National Human Research Protections Advisory Committee; NIH: National Institutes of Health; OHRP: Office for Human Research Protections; SACHRP: Secretary's Advisory Committee on Human Research Protections.

## Competing interests

The authors declare that they have no competing interests.

## Authors' contributions

The conference and workshops were organized by LN and RLR; conference presentations and workshops were summarized by LN, DB, MG, LC, and RJL; the first and final drafts of the manuscript were prepared by RJL, MG, and RLR; all authors contributed to the manuscript and approved the final manuscript.

## Appendix 1: Respondents who contributed to the consensus discussion

Mark Bach, Dorothy Becker, Inese Beitins, Jeff Botkin, Laura Brosch, Philip Budashewitz, William Burman, Leona Cuttler, Robert Daum, Norman Fost, Myron Genel, Robert Goldstein, Catherine Gordon, Dave Gould, Gilman Grave, Carla Greenbaum, Steven Hirschfeld, Julie Kanishiro, Loretta Kopelman, Susan Kornetsky, Jennifer Kulynych, John Lantos, Richard Legro, Robert Levine, Barbara Lippe, Saul Malozowski, Jerry Menifkoff, Robert Nelson, Lynnette Nieman, Theresa O'Lonergan, Pearl O'Rourke, Ivor Pritchard, Kevin Prohaska, Alan Rogol, Daniel Rosenblum, Stephen Rosenfeld, Robert Rosenfield, Peter Schmidt, F. Gary Toback, Sam Wells, David Wendler, Karen Winer

## Endnotes

Footnote

^1^OHRP is currently seeking public comment regarding implementation of such an appeal process [10]. Office for Human Research Protections (OHRP): Advance notice of proposed rulemaking (ANPRM), human subjects research protections: Enhancing protections for research subjects and reducing burden, delay, and ambiguity for investigators. 2011:http://www.hhs.gov/ohrp/humansubjects/anprm2011page.html
